# mTORC1-dependent suppression of autophagic activity in somatic cell nuclear transfer mouse embryos

**DOI:** 10.1530/REP-25-0338

**Published:** 2025-10-28

**Authors:** Takaki Tatebe, Dinh Quoc Pham, Atsuo Ogura, Kimiko Inoue

**Affiliations:** ^1^ RIKEN BioResource Research Center, Tsukuba, Ibaraki, Japan; ^2^ Graduate School of Science and Technology, University of Tsukuba, Tsukuba, Ibaraki, Japan; ^3^ School of Biotechnology, International University, Vietnam National University Ho Chi Minh City, Ho Chi Minh, Vietnam

**Keywords:** somatic cell nuclear transfer, autophagy, mTORC1, preimplantation embryo

## Abstract

**In brief:**

The non-genomic factors responsible for developmental arrest in SCNT embryos remain poorly understood. Using live-cell fluorescence imaging, we revealed that autophagic activity is impaired in preimplantation SCNT embryos, possibly due to ectopic activation of the mTORC1 signaling pathway, providing new insights into cytoplasmic barriers to cloning efficiency.

**Abstract:**

Activation of autophagy after fertilization is essential for mammalian embryonic development, as it supplies embryos with nutrients and energy. Somatic cell nuclear transfer (SCNT) embryos frequently exhibit developmental arrest, largely because of incomplete genomic reprogramming; however, the role of non-genomic factors remains unclear. Here, we investigated autophagy dynamics in mouse SCNT embryos using immunostaining and live-cell fluorescence imaging. In fertilized embryos, autophagy increased markedly from the late 2-cell stage and peaked at the morula stage. SCNT embryos followed a similar timeline but consistently showed reduced autophagic activity. Notably, the autophagic activity levels varied among SCNT embryos and positively correlated with their developmental potential. Attempts to enhance genomic reprogramming, including the removal of somatic histone methylation, did not restore autophagy. Instead, transcriptome analysis revealed ectopic activation of mTORC1 signaling as a likely cause of impaired autophagy. Consistently, treatment with an mTORC1 inhibitor successfully rescued autophagic activity in SCNT embryos. These findings identify a persistent autophagy defect during preimplantation development in SCNT embryos and suggest that modulation of non-genomic pathways, such as mTORC1 signaling, could improve SCNT efficiency.

## Introduction

Somatic cell nuclear transfer (SCNT) is the sole reproductive technique to produce individuals from a single somatic cell. Induced pluripotent stem cells (iPSCs) can also give rise to offspring; however, being merely pluripotent, the production of chimeric embryos is required. The first successful mammalian SCNT was achieved in sheep in 1997, and since then, more than 20 species have been successfully produced with the SCNT technique (Wilmut *et al.* 1997, Inoue 2023). Because SCNT can produce genetically identical individuals to donor somatic cells, this technology is expected to contribute to a variety of practical applications, including agriculture, the pharmaceutical industry, regenerative medicine, and conservation of endangered animal species (Inoue 2023, Cowl *et al.* 2024). However, SCNT embryos exhibit much lower developmental efficiency compared to normally fertilized embryos, and improvement of their birth rate is required for their practical use.

Mice have been extensively used for studying SCNT-specific developmental properties because of their well-defined genetic backgrounds (Inoue 2023). Previous studies have mainly focused on the genomic reprogramming of the transferred nucleus to correct the epigenetic aberrations in SCNT embryos. These approaches include treatment with histone deacetylase inhibitors (Kishigami *et al.* 2006, Inoue *et al.* 2015, Kamimura *et al.* 2021, Yang *et al.* 2021) to induce global histone acetylation and inhibition of the histone methyltransferase G9a or overexpression of histone demethylase *Kdm4d* to remove somatic cell-derived histone H3 lysine 9 trimethylation (H3K9me3) (Matoba *et al.* 2014, 2024). In addition, misregulation of imprinted genes in SCNT embryos has been addressed through genetic modifications or gene silencing to correct their expression pattern during pre- and post-implantation development (Inoue *et al.* 2010, Matoba *et al.* 2011, 2018, Inoue *et al.* 2020, Wang *et al.* 2020, Xie *et al.* 2022). However, our understanding of how cytoplasmic components, such as intracellular proteins and organelles, contribute to the reduced development of SCNT embryos remains limited.

In early fertilized embryos, large-scale protein degradation occurs during maternal–zygotic transition in association with global histone modification and zygotic genome activation (ZGA) (Aoki 2022, Satouh & Sato 2023, Kojima *et al.* 2025). The degradation of proteins and organelles in eukaryotic cells takes place through two major pathways: the ubiquitin-proteasome system and the autophagy-lysosome system (Pohl & Dikic 2019). Autophagy (and macroautophagy) is a cellular pathway that degrades cytoplasmic components to provide amino acids and energy required for further development (Mizushima *et al.* 2008). Autophagy is induced by cellular stresses such as amino acid starvation or reduced ATP levels. In this process, the Ulk1 complex forms a lipid bilayer, which engulfs proteins and other substances to form an autophagosome (Kroemer *et al.* 2010). Autophagosomes subsequently fuse with lysosomes to form autolysosomes, where hydrolases degrade substrates into amino acids, and resultant metabolites are recycled as cytosolic components (Klionsky *et al.* 2021). In mouse embryos, autophagic activity is observed transiently after fertilization and reappears at the late 2-cell stage. During maternal-zygotic transition, autophagy plays a pivotal role in degrading maternal RNAs and proteins to provide the resources for zygotic synthesis. In particular, embryos lacking ATG5, a key component for autophagosome formation, exhibit developmental arrest at the 4- to 8-cell stage accompanied by reduced protein synthesis, indicating that autophagy activation from the late 2-cell stage onwards is essential for early embryonic development (Tsukamoto *et al.* 2008). It is assumed that autophagy from the 2-cell stage onward is also crucial for the development of early SCNT embryos, but until now, studies with SCNT embryos have focused only on the 1-cell stage, leaving autophagy at the later stages largely unexplored (Shen *et al.* 2015).

In this study, we sought to characterize the stage-dependent autophagy dynamics in SCNT embryos throughout the entire preimplantation period. To this end, we employed a recently developed autophagic flux probe to quantitatively assess the transition of autophagic activity using live imaging. By tracking autophagy dynamics in SCNT embryos, we were able to monitor autophagic activity in real-time and compare it with that of normally developing fertilized embryos. This study showed that autophagic activity at the 4-cell stage may serve as an indicator of SCNT embryo quality. Furthermore, SCNT embryos exhibited lower autophagic activity than fertilized embryos, accompanied by upregulation of gene clusters associated with mTORC1 signaling, a pathway known to suppress autophagy (Liu & Sabatini 2020). Our study suggests that SCNT embryos differ from normally fertilized embryos not only in their epigenetic status and gene expression profiles but also in their cytoplasmic environment, offering a new insight into the molecular basis of SCNT technology.

## Materials and methods

### Animals

All animal experiments were approved by the Animal Experimentation Committees at the RIKEN Tsukuba Institute (T2024-004) and were performed in accordance with the committees’ guidelines. Animals were housed under controlled temperature (24 ± 1°C), humidity (55% ± 2%), and lighting conditions (daily light period, 07:00 h–21:00 h). They were maintained under specific pathogen-free conditions and provided with food and water *ad libitum*.

### *In vitro* fertilization

(C57BL/6N × DBA2) F1 (BDF1) (Japan SLC, Japan) female mice at 8–12 weeks of age were superovulated by injecting 7.5 IU equine chorionic gonadotropin (eCG) (PMS 1000, Nippon Zenyaku Kogyo Co., Ltd, Japan), followed 46–52 h later by injection of 7.5 IU human chorionic gonadotropin (hCG) (GONATROPIN, ASKA Pharmaceutical Co., Ltd, Japan). Female mice were euthanized by cervical dislocation 16 h after the hCG injection, and cumulus–oocyte complexes were collected from the ampullae of excised oviducts and placed in 80 μL droplets of human tubal fluid medium (Quinn *et al.* 1985). Spermatozoa were collected from the cauda epididymis of adult C57BL/6N and BDF1 male mice (Japan SLC) and preincubated for at least 50 min in a 200 μL droplet of human tubal fluid. After preincubation, 1–2 μL of the sperm suspension was added to the drops of oocyte culture. At 3 h post-insemination (hpi), morphologically normal fertilized embryos were collected and cultured in CZB (Chatot *et al.* 1990). All incubations were performed at 37°C under 5% CO_2_ in air.

### Somatic cell nuclear transfer

Nuclear transfer was performed following a previous report (Ogura 2017). Cumulus cells used as nuclear donors were collected from BDF1 female mice. Recipient oocytes were collected from BDF1 female mice following superovulation as above and placed in CZB droplets containing 0.1% bovine testicular hyaluronidase (385931, Merck, Germany) to remove cumulus cells. The cumulus-free oocytes were enucleated in Hepes-buffered CZB containing 7.5 μg/mL cytochalasin B (250233, Merck) using a Piezo-driven micromanipulator (PMM-150FU, Prime Tech, Japan). After being cultured in fresh CZB for 1 h, the enucleated oocytes were injected with cumulus cell nuclei in Hepes-buffered CZB at room temperature. After 1 h of culture, the injected oocytes were activated by Ca^2+^-free CZB containing 3 mM strontium chloride, 5 μg/mL cytochalasin B, and 50 nM TSA (T8552-1MG, Merck) for 1 h. Continuously, the reconstructed oocytes were cultured in CZB containing 5 μg/mL cytochalasin B and TSA for 5 h. They were further cultured in CZB containing TSA for 2 h. After washing, embryos were cultured in CZB at 37°C under 5% CO_2_ in air. For *Kdm4d* SCNT embryos, 10 pL of 1,500 ng/μL mouse *Kdm4d* mRNA was injected into non-TSA-treated embryos at 5–6 h post-activation (hpa).

### DNA vector construction

pcDNA3-GFP-LC3-RFP-LC3ΔG (#RDB19804) was provided by the RIKEN BRC through the National BioResource Project of the MEXT/AMED, Japan. GFP-LC3-RFP-LC3ΔG was cloned into pcDNA3.1-EGFP-poly(A)83 (Yamagata *et al.* 2005) to give a PolyA tail to pcDNA3.1-GFP-LC3-RFP-LC3ΔG-poly(A)83 plasmid. Restriction enzymes used were Hind III (FD0504, Thermo Fisher Scientific, USA) and Not I (FD0596, Thermo Fisher Scientific), and ligation was performed using the Quick Ligation Kit (M2200, New England Biolabs, USA). The following primers were used for Sanger sequencing to verify the construct: T7_Fw 5′-TAA​TAC​GAC​TCA​CTA​TAG​GG-3′, bGH_Rev 5′-GGA​GCT​GAC​ACG​GAA​GAT-3′.

### *In vitro* transcription of mRNA

The procedure of mRNA synthesis was carried out as described in a previous study (Matoba *et al.* 2014). Briefly, the pcDNA3.1 plasmid carrying the full-length mouse Kdm4d sequence (61553, Addgene, USA) was linearized by XbaI, and it was used as a template for *in vitro* transcription using mMESSAGE mMACHINE T7 ULTRA Transcription kits (AM1345, Thermo Fisher Scientific). Autophagic flux probes were similarly linearized with Xho I and synthesized. Aliquots were stored at −80°C until use.

### Inhibitor treatment

Rapamycin (553210, Merck) was prepared as a 100 μM stock in DMSO (TS20684, Thermo Fisher Scientific), diluted to 10 nM with CZB, and applied to 24 hpa SCNT embryos.

### Immunostaining

Embryos were fixed with 4% paraformaldehyde (P6148, Merck) in phosphate-buffered saline (PBS) containing 0.1 mg/mL polyvinyl alcohol (PVA) (P8136, Merck) for 15 min at room temperature. The fixed embryos were permeabilized for 15 min by incubation with 0.1% Triton X-100. Acid Tyrode’s solution was used to remove the zona pellucida. After blocking with 1% BSA in PVA/PBS for 30 min at room temperature, they were incubated in a mixture of primary antibodies at 4°C overnight. The primary antibodies used are as follows: rabbit anti-LC3 (14600-1-AP, Proteintech, USA, 1:100), rat anti-LAMP1 (1D4B) (sc-19992, Santa Cruz Biotechnology, USA, 1:100), and rabbit anti-mTOR (7C10) (2983, Cell Signaling Technology, USA, 1:100). Following three washes with 0.1 mg/mL PVA/PBS, the samples were incubated with secondary antibodies containing goat anti-rabbit Alexa 488 (A-11008, Thermo Fisher Scientific, 1:200) and goat anti-rat Alexa 546 (A-11081, Thermo Fisher Scientific, 1:200) for 2 h at room temperature. The nuclei were co-stained with Hoechst 33342 (H342, Dojindo, Japan). The fluorescent signals were observed with a C2 confocal microscope system (Nikon, Japan) using the oil immersion lenses Plan Apochromat 60× objective (Nikon) and Plan Apochromat 100× objective (Nikon).

### Live imaging

For live imaging to measure autophagic activity, 10 pL of 50 ng/μL autophagic flux probe was injected into 6 hpi fertilized embryos or 6 hpa SCNT embryos. An all-in-one fluorescence microscope (BZ-X800, KEYENCE, Japan) was used for imaging. An incubation chamber (STXG-KIWXA20I-SET, Tokai Hit, Japan) was set at 37°C on the microscope stage, under a 5% CO_2_ mixture to maintain optimal conditions for live-cell imaging. Two-cell embryos were placed in 7 μL of CZB medium drops covered with liquid paraffin (26137-85, Nacalai Tesque, Japan) in 35-mm glass-bottomed dishes (P35G-0-14-C, MatTek Corporation, USA). Fluorescence images of three z-axis planes were acquired in 4-μm increments every 30 min during embryo culture observation, using a Plan Apochromat 20× objective (NA 0.75, BZ-PA20, KEYENCE). Green and red fluorescence were detected using GFP (OP-87763, KEYENCE) and TRITC (OP-87764, KEYENCE) filters, respectively.

### Analysis of microscopic images

Fiji software (version 2.9.0) (Schindelin *et al.* 2012) was used for image analysis and line profiling. For 3D object counting, 31 stacked images were used, each acquired 1 μm from the glass surface of an LC3-stained embryo. After background subtraction with a rolling ball radius of 1 pixel and a Gaussian blur filter with a radius of 2 pixels, the stack image was divided into six groups. A threshold was applied to each group, and 3D object counting was performed with a size limit filter of 5–10,000 pixels. Images of autophagic flux probes acquired by fluorescence live imaging were quantified for each embryo by the tracking function of BZ-H4A for BZ-X800 software. The graphs were created with Matplotlib (version 3.10.0).

### RNA-seq library preparation

Two-cell and four-cell stage embryos derived from IVF and SCNT were collected at 24 and 48 hpi or hpa, washed twice in 0.05% BSA in PBS, and flash-frozen in liquid nitrogen. Ten embryos were pooled as a single sample. Cumulus cells were isolated from BDF1 cumulus–oocyte complexes following 0.1% hyaluronidase treatment. After thawing, polyadenylated RNAs were reverse transcribed and amplified using SMART-Seq HT kits (R400748, Takara Bio, Japan). The quality of sequence libraries was examined using a 4150 TapeStation (Agilent Technologies, USA) with D5000 ScreenTape (5067-5588, Agilent Technologies). Paired-end 150 bp sequencing was performed on a NovaSeq X (embryos) or HiSeq X Ten (cumulus cells) platform (Illumina, USA).

### RNA-seq data analysis

Adapter sequences and low-quality reads were removed using fastp (version 0.22.0) (Chen *et al.* 2018). The resulting sequence reads were aligned uniquely to the mm39 mouse genome using STAR aligner software (version 2.7.10b) (Dobin *et al.* 2013) with the following parameters: ‘–alignIntronMin 20, –alignIntronMax 1000000, –alignMatesGapMax 1000000, –alignSJoverhangMin 8, –alignSJDBoverhangMin 1, –twopassMode Basic, –readFilesCommand gzcat, –outFilterType BySJout, –outFilterMultimapNmax 1, –outFilterMismatchNmax 999, –outFilterMismatchNoverReadLmax 0, –outSAMattributes NH HI NM MD, –outSAMtype BAM Unsorted, –winAnchorMultimapNmax 50’. The mapped reads were counted using featureCounts (version 2.0.1) (Liao *et al.* 2014). The expression levels of genes were calculated with normalized transcripts per kilobase million (TPM) data. DEGs were analyzed by the DESeq2 package (version 1.42.1) (Love *et al.* 2014). GO analysis was performed with the clusterProfiler package (version 4.10.1) (Yu *et al.* 2012). The heatmaps were created with ggplot2 (version 3.5.1) and pheatmap (version 1.0.12). GSEA was performed using GSEA (4.4.0) software (Subramanian *et al.* 2005). The gene expression levels in all analyzed samples in this study are shown in Table S4 (see section on Supplementary materials given at the end of the article).

### Statistical analysis

Statistical analyses were performed using R (version 4.3.3, https://www.r-project.org) and Excel (Microsoft 365). Data were analyzed by unpaired two-tailed *t*-tests. A value of *P* < 0.05 was considered statistically significant.

## Results

### Autophagy dynamics in fertilized and SCNT embryos

**Figure 1:**
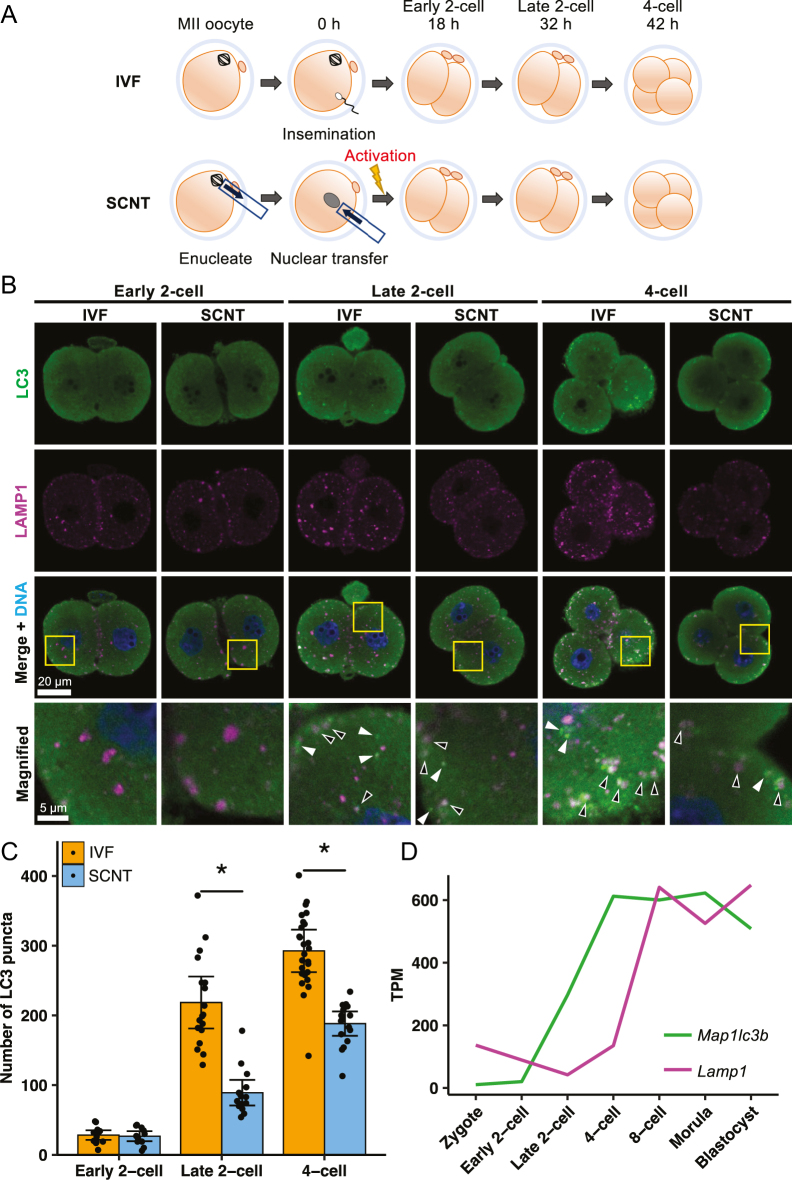
Autophagy dynamics at the 2-cell to 4-cell stage in IVF and SCNT embryos. (A) Schematic illustration of the experimental process. IVF (top) and SCNT (bottom) were used to fix early 2-cell, late 2-cell, and 4-cell samples after 18, 32, and 42 h, respectively, with 0 h of insemination or activation. (B) IVF and SCNT embryos at the 2-cell to 4-cell stage co-stained for LC3 (green) and LAMP1 (magenta). White arrowheads were autophagosomes, black arrowheads were autolysosomes, and DNA was stained with Hoechst 33342. The magnified image shows yellow squares in the bottom panels. (C) Number of LC3 puncta at the 2-cell to 4-cell stage. At least ten embryos per developmental stage were used. Significant differences were determined by a two-tailed *t*-test. *P < 0.05. Error bars indicate SEM. (D) mRNA expression profiles of *Map1lc3b* and *Lamp1* in preimplantation fertilized embryos. RNA-seq data were from publicly available sources (Deng *et al.* 2014).

First, we investigated the autophagy dynamics in mouse embryos derived from *in vitro* fertilization (IVF) and SCNT by double immunostaining for an autophagosome marker, MAP1LC3B (LC3), and the lysosome marker LAMP1. LC3 is processed to expose a C-terminal glycine, which is subsequently conjugated to phosphatidylethanolamine, producing membrane-bound LC3-II. This form is essential for the expansion and formation of the autophagosome membrane (Kabeya *et al.* 2000, Mijaljica *et al.* 2012). LC3 puncta appeared as distinct dot-like structures, reflecting the localization of LC3 on the inner and outer membranes of autophagosomes. LAMP1, a lysosomal membrane protein, interacts with the cation channel TMEM175, contributing to lysosomal pH maintenance (Eskelinen 2006, Zhang *et al.* 2023). Thus, dual labeling of LC3 and LAMP1 enabled the simultaneous monitoring of autophagosome formation and lysosomal function in early mouse embryos. In IVF embryos, LC3 signals were faint and dispersed throughout the cytoplasm, with only a small number of LC3 puncta being observed at the early 2-cell stage. From the late 2-cell stage onward, distinct dot-like puncta became clearly visible, showing a marked increase in their numbers at the late 2- and 4-cell stages (Fig. 1A and B). The average number of LC3 puncta per embryo significantly increased at the late 2-cell stage and was maintained until the 4-cell stage (Fig. 1C). We identified not only autophagosomes, represented by LC3-only puncta (white arrowhead in Fig. 1B), but also autolysosomes that were double positive or closely associated with LC3 and LAMP1 at the late 2- to 4-cell stages (black arrowhead in Fig. 1B), indicating that the normal degradation process occurred in IVF embryos (Figs 1B and S1A). According to RNA-seq analysis of preimplantation embryos (Deng *et al.* 2014), the gene expression levels of *LC3* and *Lamp1* are upregulated from the late 2-cell stage, supporting our observations above (Fig. 1D). By contrast, in SCNT embryos, whereas the number of LC3 puncta increased at the late 2- and 4-cell stages as observed in IVF embryos, it was significantly lower than in IVF embryos (Fig. 1C). In addition, fewer autolysosomes were formed in SCNT embryos compared to IVF embryos (Fig. 1B).

### SCNT embryos have low autophagic activity that cannot be rescued by improving gene expression

**Figure 2:**
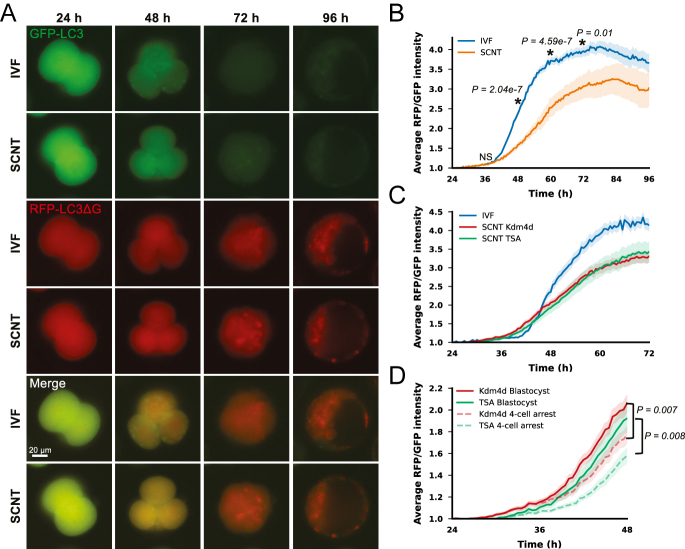
Preimplantation autophagic activity compared in IVF and SCNT embryos. (A) Representative fluorescent images of IVF and SCNT embryos at various times after fertilization or activation. From left to right, 24, 48, 72, and 96 h later were shown, IVF embryos on the upper row, and SCNT embryos on the lower row. GFP-LC3 (green), RFP-LC3ΔG (red). (B) Preimplantation autophagic activity in IVF and SCNT embryos. The *x*-axis shows the elapsed time, and the *y*-axis shows the relative RFP/GFP values for each embryo based on the 24-h time point. IVF (blue), SCNT (orange), lighter-colored areas indicate SEM. Two-tailed *t*-tests were performed at 36, 48, 60, and 72 h. IVF *n* = 35, SCNT *n* = 6. (C) Autophagic activity in IVF and SCNT embryos that were treated to enhance gene expression from 24 to 72 h after insemination or activation. The *x*-axis shows the elapsed time, and the *y*-axis shows the relative RFP/GFP values for each embryo based on the 24-h time point. IVF (blue), Kdm4d-overexpressed (red), TSA-treated (green), lighter-colored areas indicate SEM. IVF: *n* = 12, Kdm4d: *n* = 38, TSA: *n* = 18. (D) Autophagic activity up to 48 h after activation in SCNT embryos treated to enhance gene expression. Kdm4d-overexpressed (red), TSA-treated (green), lighter-colored areas indicate SEM. The broken lines indicate embryos that were subjected to the respective treatments, but embryonic development was arrested after the 4-cell stage. Two-tailed *t*-tests were performed at 48 h for each treatment group. Kdm4d arrest *n* = 22, TSA arrest *n* = 35.

Quantitative measurement of autophagic activity in living embryos was performed using an autophagic flux probe (Kaizuka *et al.* 2016) (Fig. S1A). In this system, the mRNA encoding GFP-LC3-RFP-LC3ΔG was microinjected into the cytoplasm of embryos (Fig. S1B), and the translated GFP-LC3-RFP-LC3ΔG protein was cleaved into GFP-LC3 and RFP-LC3ΔG by the protease activity of intracellular ATG4 (Fig. S1A). Because of the deletion of the C-terminal glycine of RFP-LC3ΔG, only GFP-LC3 is localized to the autophagosomes and subsequently degraded by autolysosomes. Therefore, cellular autophagic activity can be quantified by calculating the ratio of GFP-LC3 fluorescence intensity to RFP-LC3ΔG fluorescence intensity, which serves as an internal control (Fig. S1A). To quantify autophagic activity after the 2-cell stage, an mRNA probe was injected 6 h after sperm insemination (IVF) or artificial activation (SCNT), followed by live fluorescence imaging from 24 to 96 h (Fig. S1B). At 24 h, both GFP and RFP signals were strongly detected, but the GFP signal began to quench at approximately the 4-cell stage, indicating that active degradation by autolysosomes started at this stage (Fig. 2A, Video 1). Live imaging enabled real-time tracking of autophagic activity in individual embryos. In IVF embryos, autophagic activity sharply increased after 36 h and reached a peak at approximately 72–84 h (morula), followed by a slight decrease at 96 h (blastocyst) (Fig. 2B, Video 2). SCNT embryos also showed increased autophagic activity starting at 36 h, but the levels remained consistently lower than those in IVF embryos until 96 h (Fig. 2B). The difference in autophagic activity between IVF and SCNT embryos was statistically significant at 48 h (*P* < 0.0001, Fig. 2B, Video 2), despite the fluorescence signals appearing comparable in representative images. These results suggest that although SCNT embryos follow a similar autophagic timeline as IVF embryos, they exhibit overall lower activity and greater variability.

Video 1 Fluorescence live imaging and tracking of embryos injected with an autophagic flux probe. Related to Fig. 2A. Fluorescence live imaging of IVF embryos at 24 h after insemination. From left to right, BF, Merge of GFP-LC3 and RFP-LC3ΔG, and tracking regions identified from the acquired images. Scale bar indicates 20 μm. This video (https://movie-usa.glencoesoftware.com/video/10.1530/REP-25-0338/video-1) is available from the online version of the article at (https://doi.org/10.1530/REP-25-0338).
Download Video 1

Video 2 Fluorescence live imaging of autophagic flux probes in IVF and SCNT embryos. Related to Fig. 2B. Representative embryos of IVF and SCNT are shown, with time points indicating time after insemination or activation. This video (https://movie-usa.glencoesoftware.com/video/10.1530/REP-25-0338/video-2) is available from the online version of the article at (https://doi.org/10.1530/REP-25-0338).
Download Video 2

Next, we examined whether the low autophagic activity in SCNT embryos could be rescued by correcting their gene expression through epigenetic treatments widely used for mouse SCNT. We treated SCNT embryos with mRNA for a histone demethylase, *Kdm4d*, that removes H3K9me3 marks in reprogramming-resistant regions (Matoba *et al.* 2014) or with trichostatin A (TSA), a histone deacetylase inhibitor that promotes open chromatin through histone acetylation (Kishigami *et al.* 2006, Inoue *et al.* 2015, Yang *et al.* 2021). Although these treatments have been reported to improve gene expression patterns and birth rates following nuclear transfer, SCNT embryos subjected to either treatment still showed lower autophagic activity than that of IVF embryos. These results revealed that correcting gene expression profiles alone was insufficient to restore autophagic activity in SCNT embryos (Fig. 2C).

As there was large individual variation in autophagic activity among SCNT embryos, we examined whether autophagic activity was correlated with embryonic developmental ability. We compared autophagic activity between two groups of SCNT embryos: those that developed to the blastocyst stage and those that arrested at 48 h. Embryos that developed into the blastocyst stage had significantly higher autophagic activities at 48 h compared to embryos that arrested development at the 4-cell stage, irrespective of the epigenetic treatment (Fig. 2D). These results suggested that autophagic activity at 48 h (4-cell) could serve as a marker for selecting high-quality SCNT embryos.

### SCNT embryos showed significantly lower expression levels of major autophagy factor genes at the 4-cell stage

**Figure 3:**
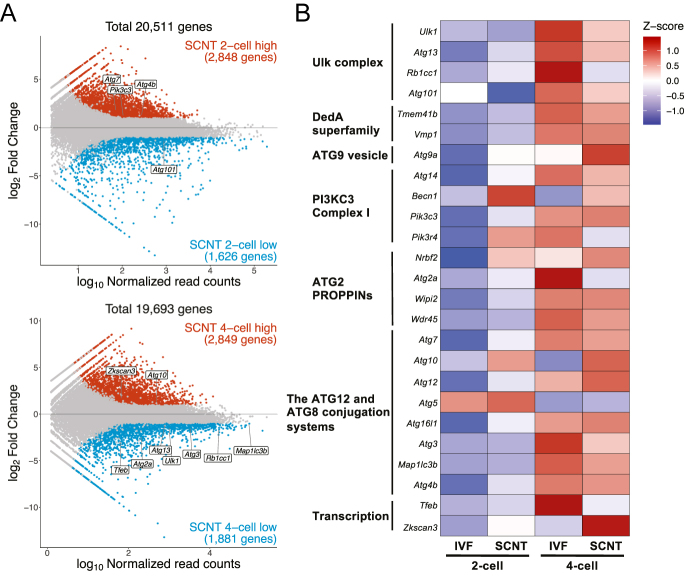
Gene expression profiles of major autophagy factors in IVF and SCNT embryos. (A) MA plot showing the ratio of gene expression levels in IVF and SCNT embryos at the 2-cell (upper) and 4-cell (lower) stages. The vertical axis shows the expression ratio, and the horizontal axis shows the number of read counts normalized by log10. Red dots indicate genes with significantly higher expression in SCNT embryos, and blue dots indicate genes with significantly lower expression in SCNT embryos. Differentially expressed genes were defined as those with an absolute log2 expression ratio ≦ 1 and an adjusted *P* value < 0.05 using DESeq2. Major autophagy factors within differentially expressed genes are indicated. (B) Heatmap of major autophagy factors at the 2-cell to 4-cell stage in IVF and SCNT embryos, with TPM values converted to Z-scores for each gene.

Our comparative live imaging analysis of autophagic activity in IVF and SCNT embryos revealed that the 4-cell stage is a critical time point that determines subsequent developmental events. Next, we performed RNA-seq analysis using 2-cell and 4-cell stage embryos to gain insight into the SCNT-specific low autophagic activity from a transcriptomic perspective. First, we identified differentially expressed genes (DEGs) between IVF and SCNT embryos at the 2-cell and 4-cell stages to determine whether major autophagy factor genes were among them (Yamamoto *et al.* 2023). At the 2-cell stage, only a few autophagy genes were found among the DEGs, but in 4-cell stage embryos, the expression levels of *Ulk1*, *Atg13*, and *Rb1cc1* (*FIP200*), components of the Ulk complex (Hara *et al.* 2008, Jung *et al.* 2009), were significantly lower in SCNT embryos. Furthermore, *Tfeb*, a key transcription factor that regulates enhanced autophagy and lysosomal gene expression (Settembre *et al.* 2011), was expressed at lower levels in SCNT embryos. In contrast, the expression of *Zkscan3*, which repressively regulates the expression of autophagy genes, was significantly higher in SCNT embryos (Figs 3A and S2A, Tables S1 and S2) (Chauhan *et al.* 2013). Gene ontology (GO) analysis of DEGs at the 4-cell stage revealed GO terms related to autophagy in downregulated genes in SCNT embryos (Fig. S2B).

Next, we evaluated the stage-dependent expression patterns of individual autophagy-related genes at the 2- cell and 4-cell stages. During the transition from the 2- to 4-cell stage in IVF embryos, most genes exhibited increased expression levels (Fig. 3B). Although SCNT embryos largely followed the gene expression patterns observed in IVF embryos, several key genes, particularly those essential for autophagosome formation, such as components of the Ulk complex and the DedA superfamily, exhibited significantly lower expression levels in SCNT embryos (Figs 3B and S2A) (Okawa *et al.* 2021). In addition, some autophagy-related genes, such as *Becn1*, *Pik3r4*, *Nrbf2*, *Atg10*, or *Atg5*, were misregulated at the 2-cell stage in SCNT embryos (Fig. 3B). Our transcriptome analysis indicates that aberrant expression of autophagy-related genes in the early preimplantation stage is likely associated with defective autophagy in SCNT embryos.

### SCNT embryos exhibit high expression of genes that enhance mTORC1

**Figure 4:**
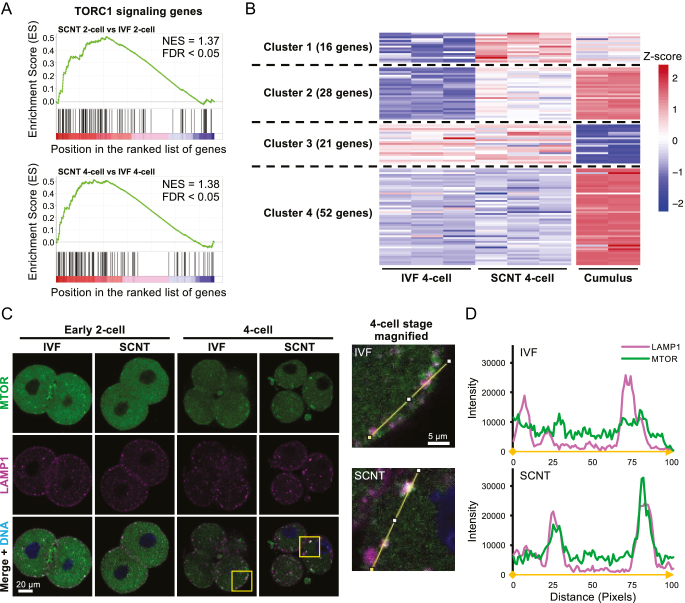
Expression of TORC1 signaling genes and intracellular dynamics of mTOR in IVF and SCNT embryos. (A) Line plots showing gene set enrichment analysis (GSEA) of TORC1 signaling (GO:0038202) for SCNT and IVF embryos. The upper column shows the 2-cell stage, and the lower column shows the 4-cell stage, with normalized enrichment score (NES) and FDR *q*-value displayed, respectively. (B) Heatmap showing gene expression at the 4-cell stage of IVF and SCNT embryos, and cumulus cells used as donor cells for SCNT, for the gene groups analyzed in A. TPM values were converted to Z-scores for each gene. The K-means method was used to divide the data into four clusters. (C) Immunostaining for mTOR (green) and the lysosomal marker LAMP1 (magenta) in IVF and SCNT embryos at the 2-cell to 4-cell stage. DNA was stained with Hoechst 33342. The magnified image shows yellow squares in the 4-cell stage. (D) Line profile showing fluorescence intensity along the yellow line drawn in the magnified image in C. The fluorescence intensity of mTOR (green) and LAMP1 (magenta) is shown at 100 pixels from the yellow point to the white point, respectively.

mTORC1 is a complex that promotes cell growth and proliferation by phosphorylating downstream substrates (Liu & Sabatini 2020). Activated mTORC1 has also been reported to inhibit autophagy through phosphorylating ULK1 and ATG13 (Hosokawa *et al.* 2009). To determine the potential involvement of mTORC1 in the reduced autophagic activity in SCNT embryos, we analyzed RNA-seq data for the genes related to the mTORC1 signaling pathway. Gene set enrichment analysis (GSEA) of TORC1 signaling genes revealed significant upregulation in SCNT embryos compared to that in IVF embryos at the 2-cell and 4-cell stages (Fig. 4A).

Next, we performed K-means clustering analysis on the mTORC1-related genes in IVF and SCNT 4-cell embryos and cumulus cells used as nuclear donors. The analysis identified four distinct clusters: clusters 1 and 2 included genes highly expressed in SCNT embryos and cumulus cells; cluster 3 was highly expressed in embryos but not in cumulus cells; and cluster 4 contained genes highly expressed only in cumulus cells (Fig. 4B, Table S3). Expression levels for representative genes enriched in clusters 1 and 2 are shown in Fig. S3. Notably, cluster 1 included several components of the Ragulator complex, *Lamtor1*, *Lamtor2*, *Lamtor3*, and *Lamtor4*, which are a pentameric protein complex that tethers the mTORC1 complex to the lysosomal surface and activates mTORC1 via interaction with Rag GTPases (Sancak *et al.* 2010, Yonehara *et al.* 2017). Cluster 2 was enriched for *Rps6kb1*, which is activated by mTORC1 (Burnett *et al.* 1998), and *Slc38a9*, an amino acid transporter-like membrane protein on lysosomes that binds to Ragulator and activates mTORC1 (Rebsamen *et al.* 2015, Wang *et al.* 2015). These results suggested that mTORC1 is activated in SCNT embryos because of higher expression of mTORC1-activating factors compared to IVF embryos. This abnormal gene activation seems to be caused by a donor cell-derived gene expression bias, as shown in the clustering pattern in Fig. 4B.

mTORC1 activation is mediated by GTP-bound Rheb on lysosomes (Sancak *et al.* 2008). To visualize the dynamics of mTORC1, we performed co-staining of mTOR and LAMP1 and evaluated the signal intensity of mTOR on lysosomes. Consistent with Wang *et al.* (2013), the fluorescence intensity of cytoplasmic mTOR was high at the 2-cell stage in both IVF and SCNT embryos, but SCNT embryos exhibited stronger mTOR signals on lysosomes compared to IVF embryos at the 4-cell stage (Fig. 4C and D). Thus, these findings indicated that mTORC1 accumulated on the lysosomes and was hyperactivated in SCNT embryos.

### Inhibition of mTORC1 in SCNT embryos increases autophagic activity

**Figure 5:**
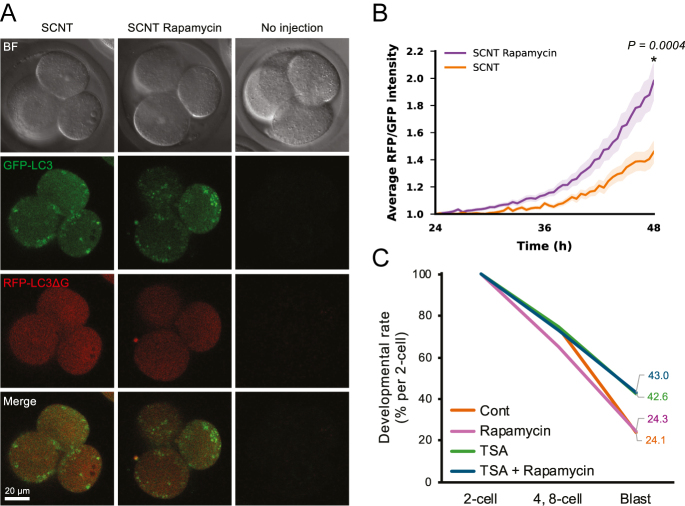
Autophagic activity of SCNT embryos treated with an mTORC1 inhibitor. (A) Representative confocal images of SCNT embryos injected with probes at the 4-cell stage. From left to right: untreated, rapamycin-treated from 24 to 48 h after activation, and probe-uninjected. GFP-LC3 (green), RFP-LC3ΔG (red). (B) Autophagic activity in rapamycin-treated and untreated SCNT embryos from 24 to 48 h after activation. Rapamycin-treated (purple), untreated (orange), colored areas indicate SEM. Two-tailed *t*-tests were performed at 48 h. SCNT rapamycin *n* = 24, SCNT *n* = 20. (C) Line chart comparing blastocyst development rates. The vertical axis is the incidence based on the 2-cell stage, and the horizontal axis is the developmental stage. Rapamycin-treated cells were analyzed 24–96 h after activation. Cont, SCNT control; TSA, TSA-treated SCNT control.

As mTORC1 was activated at the 4-cell stage in SCNT embryos, we investigated whether treatment with rapamycin, an mTORC1 inhibitor, after 24 h post-activation could activate autophagy in SCNT embryos (Fig. 5A). Previous reports showed that autophagy could be induced by rapamycin treatment in cultured cells (Blommaart *et al.* 1995, Kaizuka *et al.* 2016). When we performed an autophagic activity assay on rapamycin-treated SCNT embryos, the ratio of RFP-LC3ΔG/GFP signal intensities increased as early as 24 h post-activation. The difference was significantly evident at 48 h (*P* = 0.0004, Fig. 5B). However, continuous rapamycin treatment from 24 to 96 h post-activation did not increase the blastocyst rate of SCNT embryos, irrespective of TSA treatment (Fig. 5C). This result indicated that although rapamycin treatment enhances autophagic activity in SCNT embryos at the 4-cell stage, it has no beneficial effect on further embryonic development.

## Discussion

Autophagy is a cytoplasmic process essential for normal embryonic development. Tsukamoto *et al.* (2008) demonstrated that the complete depletion of autophagy by maternal and zygotic knockout of Atg5 results in developmental arrest at the 4- to 8-cell stage. This finding suggests that autophagy activated after ZGA, rather than maternally inherited autophagy, plays a predominant role in supporting early embryonic development. Consistent with this, we found that autophagic activity, monitored using a live-cell fluorescence system, increased rapidly at approximately 36 h after fertilization, peaked at 72–84 h, and slightly declined by 96 h. This stage-dependent pattern of autophagy closely mimicked the changes in lysosome number observed in preimplantation mouse embryos (Tsukamoto *et al.* 2013). In SCNT embryos, autophagy proceeded in a similar temporal pattern but remained consistently lower than that observed in fertilized embryos. Moreover, SCNT embryos exhibited substantial inter-embryo variability in autophagic activity during the later stages of preimplantation development. Based on this variability, we successfully identified high-quality SCNT embryos at 48 h post-activation (4-cell) using fluorescent live imaging, which reliably predicted their potential to develop to the blastocyst stage (Fig. 2D). A previous study using GFP-LC3-injected embryos also demonstrated the potential to identify high-quality embryos at the 4-cell stage (Tsukamoto *et al.* 2014). Thus, visualizing autophagic activity shortly after ZGA may aid in the classification of embryos based on their developmental potential. It would be of interest to determine whether this approach applies to other mammalian species.

In this study, we aimed to identify the cause of reduced autophagic activity in SCNT embryos. We initially hypothesized that inefficient ZGA was the primary cause, as embryonic autophagy largely depends on zygotically activated genes (Tsukamoto *et al.* 2008). Our RNA-seq transcriptome analysis revealed that SCNT embryos at the 4-cell stage exhibited lower expression of autophagy-related genes. In particular, *Tfeb*, a key transcription factor regulating autophagy and lysosome-related genes, was downregulated (Fig. 3). These findings suggest that autophagy is suppressed at the transcriptional level in SCNT embryos. However, enhancing ZGA through treatment with TSA or *Kdm4d* failed to increase autophagic activity, indicating that additional factors may be involved in the suppression of the autophagy pathway.

SCNT embryos exhibited higher expression levels of mTORC1 signaling-related genes compared to fertilized embryos. In addition, an abnormal accumulation of mTOR on lysosomes was observed at the 4-cell stage in SCNT embryos, indicating aberrant activation of the mTORC1 pathway (Fig. 4). This hyperactivation of mTORC1 may result from the persistence of mTORC1-activating components inherited from donor cumulus cells. Such ectopic activation of mTORC1 in SCNT embryos most likely leads to reduced autophagic activity, as mTOR-dependent phosphorylation of TFEB causes its retention in the cytoplasm, thereby suppressing lysosomal and autophagic functions (Roczniak-Ferguson *et al.* 2012). Consistent with this assumption, rapamycin, an mTORC1 inhibitor, effectively enhanced the autophagic activity in SCNT embryos. However, in our experimental system, rapamycin treatment with or without TSA did not improve the developmental potential of SCNT embryos (Fig. 5). This result may suggest that the reduction in autophagic activity in SCNT embryos is not significant enough to impair development or that rapamycin itself may negatively affect preimplantation development, as previously reported by Ma *et al.* (2023). Notably, our findings differ from those of a previous study in which rapamycin treatment restored autophagy shortly after activation and improved the blastocyst formation rate in SCNT embryos (Shen *et al.* 2015). That study also noted that actin polymerization inhibitors such as cytochalasin B, which are used to maintain diploidy in reconstructed embryos, interfere with autophagy. However, more recent work has shown that actin polymerization inhibitors block the accumulation, but not the formation, of intracellular membrane structures such as autophagosomes (Satouh *et al.* 2025).

In summary, quantitative analysis of autophagic activity using immunostaining and live imaging revealed that SCNT embryos exhibit impaired activation of autophagy, which is associated with reduced expression of transcription factors regulating autophagy-related genes. Notably, elevated mTORC1 activity appears to be a primary cause of this suppression, as treatment with rapamycin, an mTORC1 inhibitor, restored autophagic activity in SCNT embryos. We identified a persistent defect in autophagy during preimplantation development in SCNT embryos and propose that the modulation of non-genomic pathways, such as mTORC1 signaling, may offer a strategy to improve SCNT efficiency.

## Supplementary materials











## Declaration of interest

The authors declare that there is no conflict of interest that could be perceived as prejudicing the impartiality of the work reported.

## Funding

This work was supported by KAKENHI (19H05758 and 24K21266 to AO; 23K23799, 22K19259, and 23H04956 to KI), JST SPRING (JPMJSP2124, TT), RIKEN Junior Research Associate Program (TT), International Program Associate (DQP), and The Naito Foundation (KI).

## Author contribution statement

TT, AO, and KI designed the study. TT produced SCNT and IVF embryos and analyzed their autophagic activity using immunostaining and live imaging. TT also performed RNA-seq data analysis. TT and DQP produced SCNT embryos for the inhibitor treatment experiments.

## Data availability

The accession number for the RNA-seq datasets reported in this paper is GSE306587.
