# Supplementary material for: mTORC1-dependent suppression of autophagic activity in somatic cell nuclear transfer mouse embryos
Source: Reproduction. 2025 Oct 28;170(6):e250338. doi: 10.1530/REP-25-0338 (PMC12910570; doi:10.1530/REP-25-0338)
Supplement: Supplementary file 3 [file supplementary_materials.pdf]

Supplementary materials

mTOR1C-dependent suppression of autophagic activity in somatic cell nuclear transfer mouse embryos

Takaki Tatebe, Dinh Quoc Pham, Atsuo Ogura, Kimiko Inoue

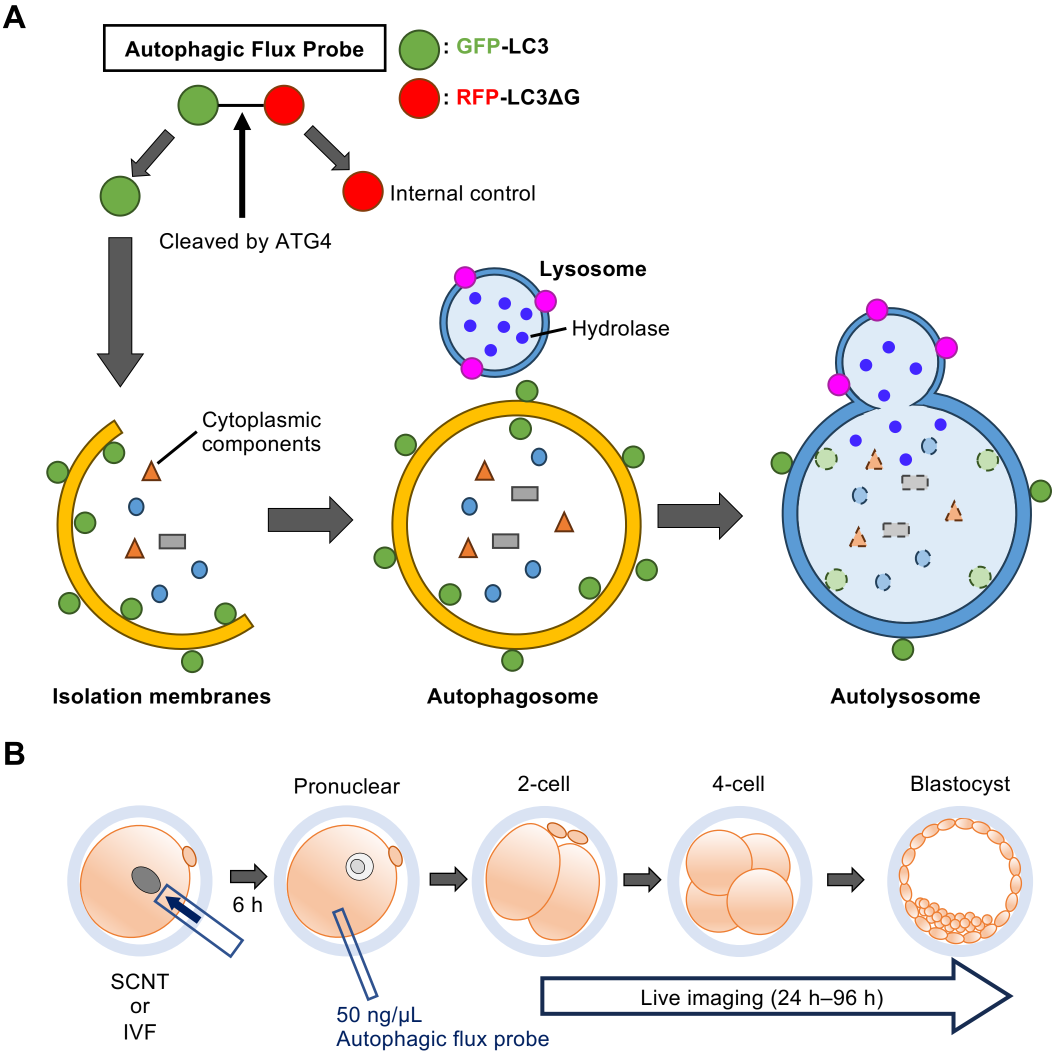

A
Autophagic Flux Probe
: GFP-LC3
: RFP-LC3ΔG
Internal control
Cleaved by ATG4
Lysosome
Hydrolase
Cytoplasmic components
Isolation membranes
Autophagosome
Autolysosome
B
Pronuclear
2-cell
4-cell
Blastocyst
6 h
SCNT
or
IVF
50 ng/μL
Autophagic flux probe
Live imaging (24 h–96 h)

Fig. S1. Schematic diagram of autophagic flux probe mechanism and experimental flow.

(A) Schematic diagram of the dynamics of autophagic flux probe in the cell. The expressed probe is cleaved into GFP-LC3 and RFP-LC3ΔG by the protease activity of endogenous ATP4, and GFP-LC3 is integrated into autophagosomes and degraded by autolysosomes, while RFP-LC3ΔG remains in the cytosol as an inner control, allowing for the quantification of autophagic activity. (B) Schematic diagram of the experiment to assay autophagic activity using autophagic flux probe. SCNT or IVF embryos were injected with 50 ng/μL probe 6 h after the start time of activation or insemination, respectively. Afterwards, the embryos were incubated overnight and transferred to an imaging chamber to perform fluorescence live imaging from 24 h to 96 h after activation or insemination.

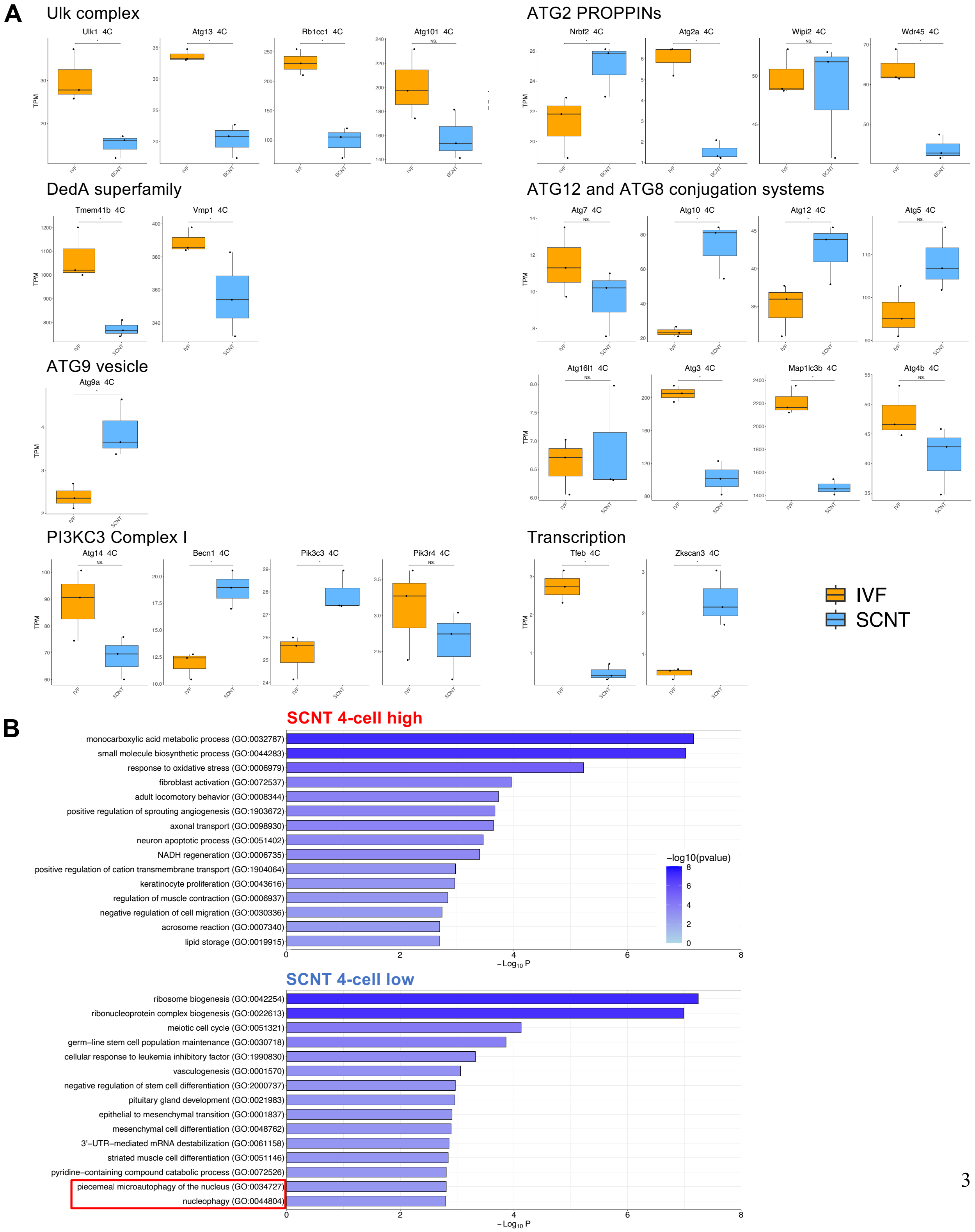
A
Ulk complex
Ulk1 4C
Atg13 4C
Rb1cc1 4C
Atg101 4C
ATG2 PROPPINs
Nrbf2 4C
Atg2a 4C
Wipi2 4C
Wdr45 4C
DedA superfamily
Tmem41b 4C
Vmp1 4C
ATG12 and ATG8 conjugation systems
Atg7 4C
Atg10 4C
Atg12 4C
Atg5 4C
ATG9 vesicle
Atg9a 4C
Atg16l1 4C
Atg3 4C
Map1lc3b 4C
Atg4b 4C
PI3KC3 Complex I
Atg14 4C
Becn1 4C
Pik3c3 4C
Pik3r4 4C
Transcription
Tfeb 4C
Zkscan3 4C
TPM
IVF
SCNT
NS.
B
SCNT 4-cell high
monocarboxylic acid metabolic process (GO:0032787)
small molecule biosynthetic process (GO:0044283)
response to oxidative stress (GO:0006979)
fibroblast activation (GO:0072537)
adult locomotory behavior (GO:0008344)
positive regulation of sprouting angiogenesis (GO:1903672)
axonal transport (GO:0098930)
neuron apoptotic process (GO:0051402)
NADH regeneration (GO:0006735)
positive regulation of cation transmembrane transport (GO:1904064)
keratinocyte proliferation (GO:0043616)
regulation of muscle contraction (GO:0006937)
negative regulation of cell migration (GO:0030336)
acrosome reaction (GO:0007340)
lipid storage (GO:0019915)
−log10(pvalue)
−Log10 P
SCNT 4-cell low
ribosome biogenesis (GO:0042254)
ribonucleoprotein complex biogenesis (GO:0022613)
meiotic cell cycle (GO:0051321)
germ−line stem cell population maintenance (GO:0030718)
cellular response to leukemia inhibitory factor (GO:1990830)
vasculogenesis (GO:0001570)
negative regulation of stem cell differentiation (GO:2000737)
pituitary gland development (GO:0021983)
epithelial to mesenchymal transition (GO:0001837)
mesenchymal cell differentiation (GO:0048762)
3'−UTR−mediated mRNA destabilization (GO:0061158)
striated muscle cell differentiation (GO:0051146)
pyridine−containing compound catabolic process (GO:0072526)
piecemeal microautophagy of the nucleus (GO:0034727)
nucleophagy (GO:0044804)
−Log10 P

Fig. S2. GO analysis and gene expression comparison of major autophagy factors in IVF and SCNT embryos at the 4-cell stage.

(A) Box plot showing expression levels of major autophagy factors at the 4-cell stage of IVF and SCNT embryos. P values were calculated by two-tailed t-tests, and those with $p < 0.05$ are indicated by *.(B) Gene ontology (GO) analysis of genes with upper and lower expression in SCNT embryos at the 4-cell stage.

Cluster 1

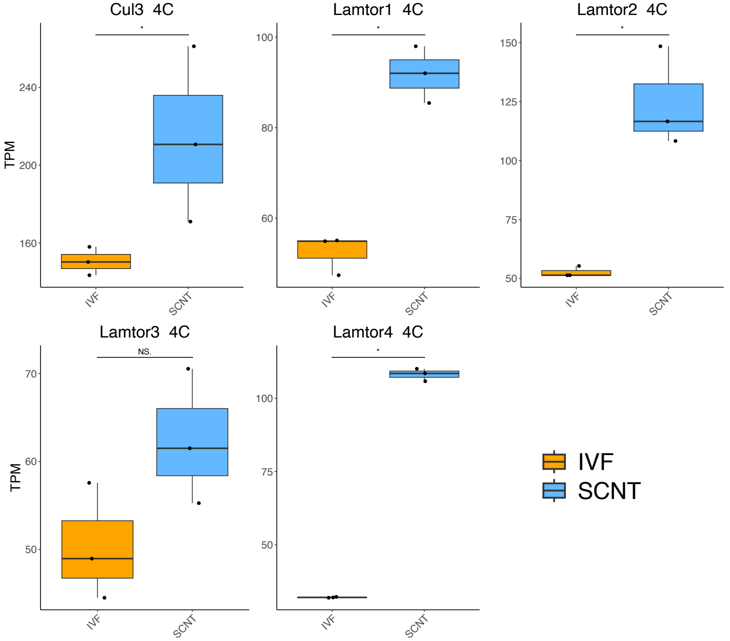

Cluster 2

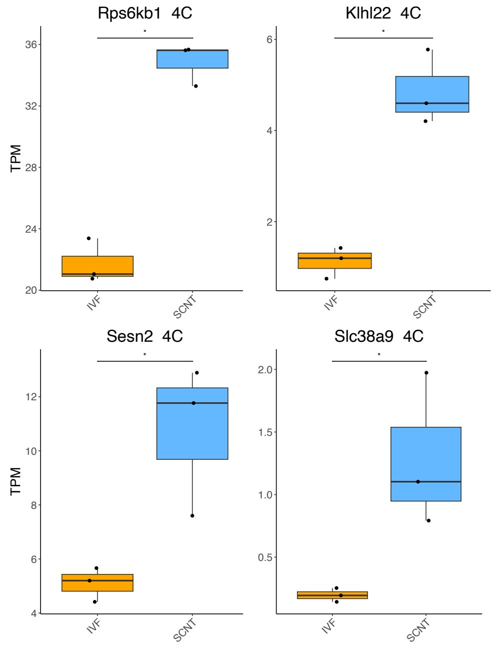

Fig. S3. Comparison of gene expression between IVF and SCNT embryos for representative genes in the clusters.

Box plot comparing gene expression levels in IVF and SCNT embryos at the 4-cell stage for representative genes in clusters 1 and 2, as shown in Fig. 5B. P values were calculated by two-tailed t-tests, and those with $p < 0.05$ are indicated by *.
